# Clinical and Epidemiologic Characteristics of Hospitalized Patients with 2009 H1N1 Influenza Infection

**DOI:** 10.1155/2012/603989

**Published:** 2012-04-23

**Authors:** Saygin Nayman Alpat, Gaye Usluer, Ilhan Ozgunes, Elif Doyuk Kartal, Nurettin Erben

**Affiliations:** Department of Clinical Microbiology and Infectious Diseases, Faculty of Medicine, Eskisehir Osmangazi University, 26480 Eskisehir, Turkey

## Abstract

*Objective*. 2009 H1N1 virus is a new virus that was firstly detected in April 2009. This virus spreads from human to human and causes a worldwide disease. This paper aimed to review the clinical and epidemiological properties of patients with 2009 H1N1 influenza who were hospitalized and monitored at Eskisehir Osmangazi University Faculty of Medicine Hospital. *Setting*. A 1000-bed teaching hospital in Eskisehir, Turkey. *Patients-Methods*. Between 05 November 2009–01 February 2010, 106 patients with 2009 H1N1 influenza, who were hospitalized, were prospectively evaluated. *Results*. Out of 106 patients who were hospitalized and monitored, 99 (93.4%) had fever, 86 (81.1%) had cough, 48 (45.3%) had shortness of breath, 47 (44.3%) had sore throat, 38 (35.8%) had body pain, 30 (28.3%) had rhinorrhea, 17 (16%) had vomiting, 15 (14.2%) had headache, and 14 (13.2%) had diarrhea. When the patients were examined in terms of risk factors for severe disease, 83 (78.3%) patients had at least one risk factor. During clinical monitoring, pneumonia was the most frequent complication with a rate of 66%. While 47.2% of the patients were monitored in intensive care unit, 34% of them required mechanical ventilation support. *Conclusion*. Patients with 2009 H1N1 influenza, who were hospitalized and monitored, should be carefully monitored and treated.

## 1. Introduction

2009 H1N1 virus is a new virus that was firstly detected in April 2009. This virus spreads from human to human and causes a worldwide disease.

In 2009 H1N1 influenza, the symptoms include fever, tremor, rhinorrhea, cough, chest pain, body pain, headache, tiredness, diarrhea, and vomiting [1]. For 2009 H1N1 influenza infection, chronic lung disease, immunosuppression, and pregnancy were detected as risk factors for severe disease [2–4]. Throughout the pandemic period, a majority of the patients, who were below 2 years, above 65 years, and had underlying disease, were hospitalized and monitored [5, 6].

This study aimed to review the clinical and epidemiological properties of patients with 2009 H1N1 influenza who were hospitalized and monitored at Eskisehir Osmangazi University, Faculty of Medicine Hospital.

## 2. Materials and Methods

Between 05 November 2009–01 February 2010, 106 patients with 2009 H1N1 influenza, who were hospitalized and monitored at Eskisehir Osmangazi University, School of Medicine Hospital, were prospectively evaluated. The patients' demographics, complaints related to admission to hospital, duration of disease, physical examination findings, laboratory and radiological findings, name of the clinic where monitoring was performed, underlying diseases (age, chronic pulmonary, cardiovascular, renal, hepatic, hematologic, metabolic disease, neuromuscular disease, taking immunosuppressive therapy, pregnancy, obesity), complications developed, and prognosis were recorded in the patient recruitment forms prepared.

Respiratory distress, tachypnea, chest pain, confusion, persistent vomiting, worsening of general condition, and fever lasting more than three days were considered as a sign of serious illness, and these patients were hospitalized.

Criteria for hospitalization in patients with, in addition, persistent hypoxemia, hemodynamic instability, sepsis and shock findings, if any of the planned admission to intensive care unit patients. In-patients, patients without evidence of improvement in spite of antiviral therapy and supportive, progressive pulmonary infiltrate, persistent hypoxemia (SpO2 < 92%), progressive hypercapnia, hemodynamic deterioration, signs of sepsis and shock in the case planned to be taken to intensive care unit patients.

Two thousand and nine H1N1 virus definitions by the World Health Organization, a national laboratory (Refik Saydam Hygiene Center) sent with the primary probe and positive controls, “Swine Influenza PCR Testing Kit,” using “real-time reverse transcriptase polymerase chain reaction” was performed by analysis.

Statistical analysis, chisquare test, and Mann-Whitney *U* test were used.

## 3. Results

Between 05 November 2009–01 February 2010, a total of 106 patients were hospitalized and monitored. The demographics of the patients are given in Table 1. None of the patients had a history of vaccination against 2009 H1N1 virus.

 Considering the provinces where the patients came from; 78 (73.6%) of the patients were from Eskisehir, 17 (16%) from Bilecik, 5 from (4.7%) Kutahya, 2 (1.9%) from Afyon, and 1 (0.9%) each from Balikesir, Bursa, Istanbul, and Usak. 

**Figure 1:**
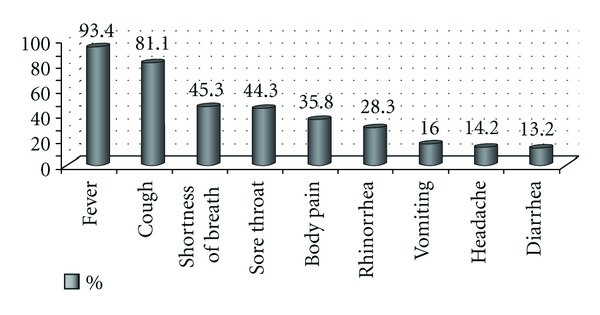
Complaints of the patients who presented to the hospital.

When the complaints of the patients who presented to the hospital were evaluated, out of 106 patients who were hospitalized and monitored, 99 (93.4%) had fever, 86 (81.1%) had cough, 48 (45.3%) had shortness of breath, 47 (44.3%) had sore throat, 38 (35.8%) had body pain, 30 (28.3%) had rhinorrhea, 17 (16%) had vomiting, 15 (14.2%) had headache, and 14 (13.2%) had diarrhea (Figure 1). 

**Figure 2:**
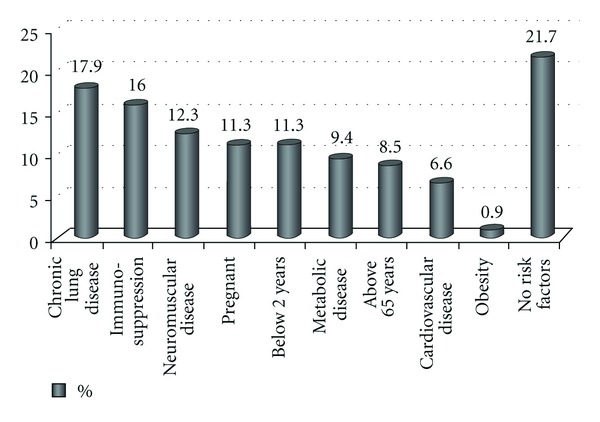
Risk factors for severe disease in patients.

When all patients were examined in terms of risk factors for severe disease, 83 (78.3%) patients had at least one risk factor. 19 (17.9%) of the patients had chronic lung disease, 17 (16%) had immunosuppression, 13 (12.3%) had neuromuscular disease, 12 (11.3%) were pregnant, 12 (11.3%) were below 2 years, 10 had (9.4%) metabolic disease, 9 (8.5%) were above 65 years, 7 (6.6%) had cardiovascular disease, 1 (0.9%) had obesity (Figure 2). 3 (25%) of 12 monitored pregnant women developed complication of pneumonia. None of them required mechanical ventilation and no mortality was observed. 

**Figure 3:**
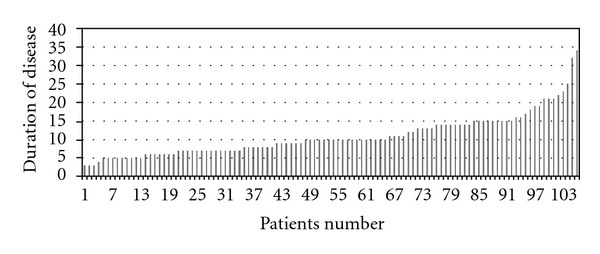
Duration of disease in patients.

In patients, duration of disease ranged between 3–34 days (10.9 ± 5.6 days on average) (Figure 3). In 106 patients, who were hospitalized and monitored due to H1N1, virus identification was performed at National Influenza Laboratory with the analysis of real-time reverse transcriptase polymerase chain reaction. The complications, which occurred during clinical monitoring of the patients, are given in Table 2. In 16 of 70 cases of pneumonia, bacterial pneumonia was considered. Of these patients, blood and sputum cultures were negative. In Patients with suspected bacterial pneumonia, empiric treatment, were started according to guidelines. 

In 106 patients with 2009 H1N1 influenza who were hospitalized, 55 (51.9%) were monitored in clinics of infectious diseases and 1 (0.9%) in oncology. A total of 50 (47.2%) patients consisting of 27 (25.5%) patients were monitored in pediatrics, 19 (17.9%) in thoracic diseases, 3 (2.8%) in internal diseases, and 1 (0.9%) in general surgery intensive care units. During intensive care monitoring, 17 (34%) patients required ventilation support associated with respiratory failure. 7 (41.2%) of them were monitored with mechanical ventilator, 6 (35.3%) were with noninvasive mechanical ventilator, and 4 (23.5%) were with noninvasive mechanical ventilator and then with mechanical ventilator. Distribution of these patients per intensive care units is given in Table 3. 

Oseltamivir treatment was administered to all patients for 5 days. 8 of 106 patients (7.5%) with 2009 H1N1 influenza died. All deceased patients were those confirmed in terms of 2009 H1N1 virus by the laboratory. 5 of 7 patients (71.4%), who were monitored with mechanical ventilator, and 2 of 4 patients (50%), who were monitored with noninvasive mechanical ventilator and then with mechanical ventilator, died; on the other hand, all patients, who were monitored with only non-invasive mechanical ventilator, recovered. To be an underlying risk factor, there was no statistically significant relationship between the need of mechanical ventilation and death. 

Distribution per age for the deaths in patients with 2009 H1N1 influenza is given in Table 4. 

All deceased patients had underlying disease that posed risk (Table 5). 

## 4. Discussıon

2009 H1N1 virus causes a disease at levels varying from mild to severe. While no treatment is required for some patients, hospitalization is required for some of them [1]. 

Considering the conducted studies, 95% of the patients with 2009 H1N1 influenza consist of patients below 50 years [2, 7]. In our patients with 2009 H1N1 influenza, who were hospitalized and monitored, mean age was detected to be 31.7 similar to the mean ages stated in different studies [8–10]. 

In hospitalized patients, the symptoms, which were detected to be typical, were as follows: fever (95%), cough (88%), shortness of breath (60%), tiredness (43%), rhinorrhea (38%), chest pain (31%), headache (34%), and myalgia (36%). Gastrointestinal complaints such as vomiting and diarrhea are seen at a lower rate [11]. Top three complaints of the patients who presented to the hospital included fever at a rate of 93.4%, cough at 81.1%, and shortness of breath at 45.3%. These complaints were followed, respectively, by sore throat, body pain, rhinorrhea, vomiting, headache, and diarrhea. 

The following are considered to be risk factors for severe complications for 2009 H1N1 influenza infection: age above 65, age below 2, pregnancy, and chronic diseases [1]. In different studies, in patients with 2009 H1N1 influenza, underlying disease was reported to be at a rate of 68–73% [10, 11]. When our patients were examined for underlying diseases which are possible risk factors, 78.3% of the patients had at least one risk factor. Chronic lung disease was detected in 17.9% of our patients, immunosuppression in 16%, neuromuscular disease in 12.3%, pregnancy in 11.3%, metabolic disease in 9.4%, cardiovascular disease in 6.6%, and obesity in 0.9%. While 11.3% of the patients consisted of those below 2 years, 8.5% of them comprised patients above 65 years. Pregnant women are under high risk in terms of morbidity and mortality associated with 2009 H1N1 influenza infection. In the studies conducted, abortus, birth defect, and preterm labor cases are reported [11–18]. 12 pregnant women were hospitalized and monitored in our hospital throughout pandemic period. No complication was seen except for pneumonia that developed in 3 patients. All patients recovered and discharged; no problem was experienced during their pregnancy followup. 

The complications frequently reported for 2009 H1N1 influenza infection were pneumonia, bacterial coinfection, and exacerbation of underlying disease [19]. In our patients during clinical monitoring, pneumonia was the most frequent complication with a rate of 66%. 

In the study conducted by Louie et al. [10], 31% of the patients with 2009 H1N1 influenza, who were hospitalized and monitored, are reported to require stay in intensive care, and 65% of them required mechanical ventilation. Whereas in the study conducted by Riquelme et al. [19], rate of stay in intensive care is reported to be 22%, and rate of patients who require mechanical ventilation is reported to be 68%. While 47.2% of our patients were monitored in intensive care unit, 34% of them required mechanical ventilation support. Contrary to the patients reported in studies of the literature, in our hospitalized patients with 2009 H1N1 influenza, it is observed that while indication of stay in intensive care is higher, requirement for mechanical ventilator is lower. 

In our patients with 2009 H1N1 influenza, mortality rate is at similar rates with other studies in the literature [10, 11]. In a study conducted by Jain et al., underlying disease is reported at a rate of 68% in patients who present a mortal course [11]. In our case series, all patients, who presented a mortal course, had underlying disease. 

## 5. Conclusion

For 2009 H1N1 influenza infection, frequent presence of respiratory failure and requirement of mechanical ventilation in patients who needed hospitalization and developed pneumonia is notable. Unlike seasonal influenza, severe course, which is present also outside the risk groups, is noteworthy. Therefore, patients with 2009 H1N1 influenza, who were hospitalized and monitored, should be carefully monitored and treated.

## Figures and Tables

**Table 1:** Demographics of patients with 2009 H1N1 influenza.

	Number of patients	%
Gender		
Male	45	42.5
Female	61	57.5

Total	106	100

Age group (years)		
below 1 year	7	6.6
1–4	9	8.5
5–14	10	9.4
15–24	14	13.2
25–44	40	37.7
45–64	16	15.1
above 65 years	10	9.4
Mean (SD)	31.7 (21.3)	

Total	106	100

**Table 2:** Complications seen in patients with 2009 H1N1 influenza.

Complication	Number of patients	%
Pneumonia	70	66
Seizure	2	1.89
Hemoptysis	1	0.94
Pleural effusion	1	0.94
Pneumothorax	1	0.94
Pericarditis	1	0.94
Neutropenia	1	0.94

Total	77	72.6

**Table 3:** Distribution of patients, who received ventilator support, by the clinics where they were monitored.

	Number of patients	%
Mechanical ventilator		
Thoracic diseases intensive care	5	71.4
Pediatrics intensive care	1	14.3
Internal diseases intensive care	1	14.3

Total	7	100

Noninvasive mechanical ventilator		
Thoracic diseases intensive care	3	50
Pediatrics intensive care	3	50

Total	6	100

Noninvasive mechanical ventilator + mechanical ventilator		
Thoracic diseases intensive care	3	75
General surgery intensive care	1	25

Total	4	100

**Table 4:** Distribution per age for the deaths in patients.

Age group (years)	Number of patients	Number of deaths (%)
below 1 year	7	1 (14.3)
1–4	9	0 (0)
5–14	10	0 (0)
15–24	14	2 (14.3)
25–44	40	4 (10)
45–64	16	0 (0)
above 65 years	10	1 (10)

**Table 5:** Underlying diseases in deceased patients.

Underlying disease	Number of deceased patients
Immunosuppression	2
Neuromuscular disease	1
Metabolic disease	1
Cardiovascular disease	1
Obesity	1
above 65 years + metabolic disease	1
below 2 years + neuromuscular disease	1

Total	8
